# Automatic collateral quantification in acute ischemic stroke using U^2^-net

**DOI:** 10.3389/fneur.2025.1502382

**Published:** 2025-05-12

**Authors:** Qingqing Lu, Hongyi Chen, Junyan Fu, Xiaodong Zheng, Yiren Xu, Yuning Pan

**Affiliations:** ^1^Department of Radiology, The First Affiliated Hospital of Ningbo University, Ningbo University, Ningbo, China; ^2^Department of Radiology, State Key Laboratory of Medical Neurobiology, Huashan Hospital, Fudan University, Shanghai, China; ^3^Academy for Engineering and Technology, Fudan University, Shanghai, China

**Keywords:** quantitative collateral score, visual collateral score, acute ischemic stroke, deep learning, U^2^-Net

## Abstract

**Objectives:**

To harness the U^2^-Net deep learning framework for automated quantification of collateral circulation in acute ischemic stroke (AIS) via computed tomography angiography (CTA) images, comparing its performance against traditional visual collateral scores (vCS).

**Methods:**

A cohort of 118 confirmed AIS cases was assembled and stratified into 94 development and 24 test cases. CTA images underwent preprocessing and annotation. The U^2^-Net was trained to segment collateral vessels, yielding a quantitative collateral score (qCS) based on vessel volume ratios between affected and healthy hemispheres. Performance was assessed via Dice Similarity Coefficient (DSC), Spearman correlation, Intraclass Correlation Coefficient (ICC), and accuracy, with comparisons to vCS (Tan and Menon score) and ground truth.

**Result:**

The U^2^-Net demonstrated robust segmentation capabilities, achieving a mean DSC of 0.75 in the test set. The qCS showed a strong correlation with vCS with *ρ* ranging from 0.78 to 0.92. When compared to the more refined six-class Menon score, the qCS exhibited stronger consistency (development set: ICC = 0.83, test set: ICC = 0.93) than when compared to the four-class Tan score (development set: ICC = 0.76, test set: ICC = 0.79). In terms of classification accuracy, the AI model achieved 0.83 and 0.71 against ground truth and vCS, respectively, for four-class classification. This accuracy escalated to 0.88 and 0.83 for binary classification, emphasizing its proficiency in differentiating collateral status.

**Conclusion:**

Our U^2^-Net AI model offers a reliable, objective tool for quantifying collateral circulation in AIS. The qCS aligns well with vCS and demonstrates the feasibility of automated collateral assessment, which may enhance diagnostic accuracy and therapeutic decision-making.

## 1 Introduction

In the realm of acute ischemic stroke (AIS) management, collateral circulation stands as a pivotal determinant of clinical outcomes, serving as a crucial benchmark for assessing the feasibility of mechanical thrombectomy beyond time window (1, 2). Among the myriad of imaging modalities, computed tomography angiography (CTA) has firmly established itself as the most common method, owing to its convenience, and unparalleled temporal and spatial resolution which enables meticulous delineation of collateral vessel architecture. Despite the proliferation of CTA-based collateral scoring systems, a universally accepted and superior methodology remains elusive (3), with conventional visual scoring methods beset by notable intra-and inter-observer variability, spanning a range of 0.49 to 0.97 (4, 5). This variability, exacerbated by the heterogeneity in physician expertise and research foci within the context of clinical emergencies (6, 7), underscores the urgent need for an objective, quantifiable assessment approach.

Moreover, expediting imaging evaluation and facilitating early reperfusion holds the promise of significantly enhancing neurological recovery among AIS patients (1). However, the prerequisite manual scoring process, which necessitates time-consuming 3D reconstruction and maximum intensity projection (MIP) post-processing, can consume up to nearly 5 min per case (8), thereby exacerbating the burden on emergency imaging services. Consequently, the development of automated, objective, quantitative, and time-efficient methods for collateral circulation assessment is paramount in clinical practice.

Artificial intelligence (AI), with its unparalleled ability in self-learning feature extraction, presents a promising avenue to address this challenge. By automating both input and output processes, AI catapults diagnostic and therapeutic workflows into a new era of rapid processing speeds (9, 10). Recognizing the intricacies involved in secondary collateral vessel segmentation, the advent of the U^2^-Net architecture, renowned for its exceptional segmentation prowess, offers a compelling opportunity. Thus, this study aims to harness the power of U^2^-Net, utilizing CTA images, to automatically quantify collateral circulation and demonstrate its superior performance by comparing it with traditional visual collateral scores (vCS), thereby advancing the precision and efficiency of AIS management.

## 2 Materials and methods

### 2.1 Study population

From February 2018 to February 2021, we retrospectively reviewed AIS cases admitted to the emergency department of our hospital within 24 h of onset. This study was reviewed and approved by the local institutional review board (Approval no. 2023-no.187-01), and the need for written informed consent was waived owing to the retrospective design of the study and anonymization of the data. The inclusion criteria were as follows: (1) patients aged 18 years or older; (2) premorbid modified Rankin Scale (mRS) score ≤ 2; and (3) CTA confirmed occlusions in the intracranial internal carotid artery (ICA), M1 or proximal M2 segment of the middle cerebral artery (MCA), or tandem lesions. The exclusion criteria were as follows: (1) intracranial hemorrhage identified by non-contrast computerized tomography (NCCT); (2) occlusion of other intracranial arteries or stenosis of MCA; (3) a history of a moderate to large stroke in the contralateral hemisphere resulting in a measurable decrease in vasculature; (4) moyamoya disease; and (5) CTA images exhibiting obvious motion artifacts or improper phases.

### 2.2 Image acquisition

Images were obtained using a 64-multislice CT scanner (Discovery CT750 HD, GE Healthcare), and a NCCT of the head was performed to exclude hemorrhage initially, followed by CTA of the head and neck. Single-phase CTA was performed during the administration of 80 mL of nonionic iodine contrast agent (ioversol 320 mgI/mL, Jiangsu Hengrui Medicine Co., Ltd. China) at the rate of 4 mL/s, followed by the administration of 40 mL of saline at the same rate. Bolus tracking technology was utilized, with the monitoring point positioned at the aortic arch and a trigger threshold set at 100HU, utilizing a standard reconstruction algorithm. The scanning parameters were as follows: tube voltage 140 kV, tube current 630 mA, pitch 1.375, field of view 24 cm × 24 cm, slice thickness 0.625 mm with no slice interval, rotation time 0.5 s, reconstruction matrix 512 × 512, and a scan range extending from the aortic arch to the vertex of the skull.

### 2.3 AI-derived quantitative collateral score (qCS)

**Figure 1:**
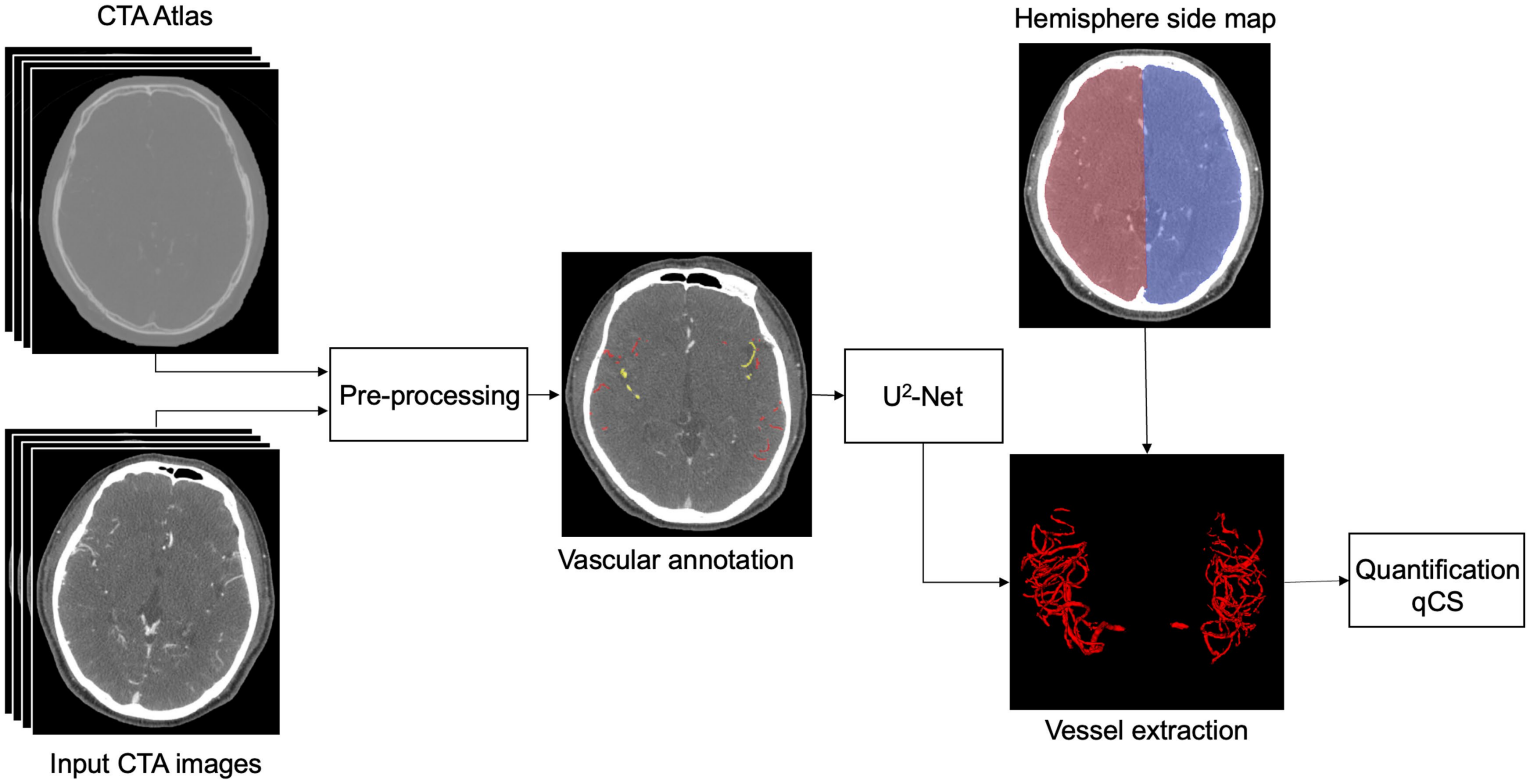
Block diagram outlining the process flow of our AI model for the quantitative assessment of cerebral collateral circulation. AI, Artificial intelligence; CTA, computed tomography angiography; qCS, quantitative collateral score.

The dataset was randomly stratified into an 80% provisional cohort for model development (with 5-fold cross-validation maintaining class distribution) and a 20% independent hold-out test cohort. The construction process of the AI-based collateral circulation quantitative assessment model is illustrated in Figure 1. A total of 280 single-phase CTA images were selected, extending from the cranial vertex to the cranial base, all of which underwent standardized preprocessing protocols comprising rigid registration and intensity normalization. Vascular annotations were created by three annotators: the first author and two experienced neuroradiologists. All annotations in both the model development cohort and hold-out test cohort underwent iterative quality control supervised by a senior cerebrovascular neuroradiologist with seven years of subspecialty experience. Arterial vessels in the MCA territories were manually segmented on consecutive axial slices using ITK-SNAP (version 3.8.0), with a uniform window width (600 HU) and level (100 HU) (see Figure 1 for examples of vascular annotation). Bilateral annotations systematically traced arteries from Circle of Willis origins to cortical branches, guided by anatomical landmarks including sylvian fissure and insular cortex, as previously mentioned (11). Differentiation between arteries and veins was achieved based on vessel courses and convergence directions.

The AI-based quantitative assessment model for collateral circulation leverages the U^2^-Net architecture (12) for vessel segmentation. U^2^-Net embodies a two-level nested U-shaped structure, where each larger U-shaped structure is filled with a well-configured residual U-block, enabling more efficient extraction of intra-stage multi-scale features and aggregation of inter-stage multi-level features. With clinical symptoms serving as the known input, the affected hemisphere can be inferred. Subsequently, based on the maps of the left and right cerebral hemispheres, the vascular volumes within the MCA territories of both hemispheres are calculated. The qCS is then defined as the percentage of vascular volume in the MCA territory of the affected hemisphere relative to that of the healthy hemisphere, formulated as follows:


$$\text{qCS}(\%)=100\times \frac{V_{\text{affected}}}{V_{\text{healthy}}\;},0\leq \text{qCS}$$

Based on the Tan score (13) thresholds (0, 50, and 100%) and clinical expertise, we systematically evaluated accuracy, sensitivity, and specificity across adjacent percentage intervals (1% increments spanning 10 percentage points around each threshold) to refine and optimize model performance (detailed results are provided in Supplementary File 1). The final thresholds of 5, 49, and 95% were selected as critical cutoffs. The results were categorized into the following binary and multi-class classifications:

Binary classification: a qCS ≤ 49% indicates poor collateral circulation, whereas a qCS > 49% signifies good collateral circulation.

Quaternary classification (0–3 point): 0 = qCS ≤ 5%; 1 = 5% < qCS ≤ 49%; 2 = 49% < qCS ≤ 95%; 3 = qCS > 95%.

Aligned with the Menon score (14) criteria and empirical observations, the thresholds were defined as:

Six-class classification (0–5 point): 0 = qCS ≤ 5%; 1 = 5% < qCS ≤ 25%; 2 = 25% < qCS ≤ 49%; 3 = 49% < qCS ≤ 75%; 4 = 75% < qCS ≤ 95%; 5 = qCS > 95%.

### 2.4 Development protocol

The U^2^-Net model was implemented using PyTorch and executed on a Linux workstation with an Intel(R) Core(TM) i9-10900X CPU and an NVIDIA Geforce GTX 3090 GPU. During 5-fold cross-validation, we performed hyperparameter tuning through grid search, evaluating learning rates (1e-3, 1e-4, 1e-5) and batch sizes (8, 16, 32). Model selection was guided by optimizing the validation Dice similarity coefficient (DSC). The final model was selected based on the fold demonstrating the highest mean validation DSC across all splits during 5-fold cross-validation. U^2^-Net performs best at 10^(−4) initial learning rate with a batch size of 8. Development employed the Adam optimizer for 500 epochs with early stopping after 20 epochs of validation DSC plateauing, alongside data augmentation strategies such as horizontal/vertical flipping (*p* = 0.5), brightness (±20%) and contrast (0.8–1.2) adjustments, and mild rotation (±10°) and zoom (0.9–1.1).

### 2.5 The performance of our AI model for collateral circulation assessment

#### 2.5.1 Compared with ground truth (GT)

For segmentation performance, the DSC was employed as the primary metric using manual segmentation as the GT. For classification performance, accuracy, F1 score, sensitivity, specificity, Matthews Correlation Coefficient (MCC), and the area under the receiver operating characteristic curve (AUC) were calculated.

#### 2.5.2 Compared with vCS

The collateral assessment was conducted using the Tan (13), Menon (14), and regional leptomeningeal collateral (rLMC) (15) scales for manual visual scoring. Regarding CTA imaging, MIP reconstructions were executed in the axial, coronal, and sagittal planes, with customized slice thicknesses: 20 mm for Tan and Menon score, and 40 mm for rLMC score, ensuring no interslice gaps. To ensure methodological rigor and avoid bias, visual scoring was conducted by an independent team comprising two seasoned radiologists (with 5-and 6-year experience of cerebrovascular disease) and one senior neuroimaging expert (with 10 years of cerebrovascular disease experience), distinct from the manual segmentation group. The radiologists independently evaluated the images under blinded conditions, unaware of the original reports, clinical histories, segmentation data and subsequent imaging findings, with the exception of the vascular occlusion location. The intraclass correlation coefficient (ICC) for consistency between the two observers in the Tan, Menon, and rLMC scores were 0.837, 0.901, and 0.928, respectively (all *p* < 0.001). In instances of disagreement between the initial two assessors, the senior expert performed an independent and definitive evaluation.

### 2.6 Statistical analysis

Continuous variables were presented as mean ± standard deviation or median (interquartile range). Categorical variables were expressed as frequency and percentage. The independent samples *t* test, Mann–Whitney *U*-test, Chi-square or Fisher’s exact test was used to compare the differences between development and testset. Spearman’s rank correlation coefficients were performed to compare qCS and vCS. ICC with absolute agreement two-way random model (single measure) were used to determine the agreement. Receiver operating characteristic (ROC) analysis were performed to compare the performance of qCS and vCS. A *p* value of <0.05 was considered statistically significant. Statistical analysis was performed using the IBM SPSS Statistics software (Version: 26.0.0.0).

## 3 Results

### 3.1 Patient demographic data

**Figure 2:**
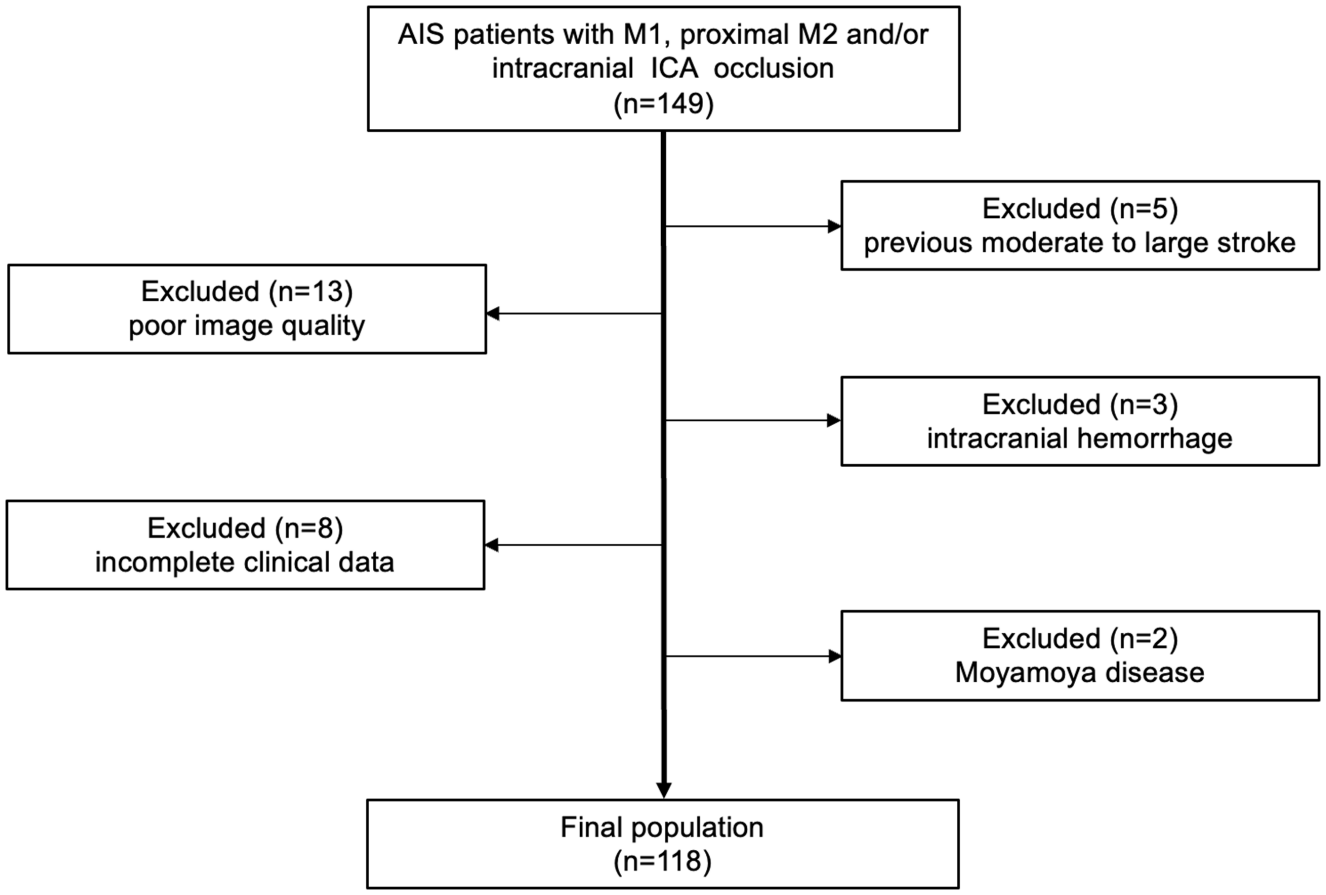
Flow chart of enrolled patients. AIS, acute ischemic stroke; ICA, internal carotid artery.

Figure 2 presents patient flowchart of the selection process and a total of 118 cases were ultimately enrolled. An 8:2 ratio yielded 94 cases for the development set and 24 for the test set. Given the relatively low number of cases with minimal collateral circulation, these were identified as a distinct subgroup and underwent separate randomization to ensure a comprehensive evaluation of model performance during test, while the remaining cases were randomly assigned in a unified manner. No statistically significant differences were observed between the development and test set in terms of age, occlusion location, Trial of Org10172 in Acute Stroke Treatment classification, baseline National Institute of Health stroke scale score, and collateral score (Table 1), indicating good clinical consistency and comparability between the two sets.

**Table 1:** Clinical characteristics of subjects.

Characteristics	All (*n* = 118)	Development set (*n* = 94)	Test set (*n* = 24)	*p* value
Age, years	73.00 (60.75, 80.00)	74.00 (60.00, 80.00)	68.00 ± 13.23	0.32
Male	78 (66.10%)	61 (64.89%)	17 (70.83%)	0.58
*Site of occlusion*				0.22
Intracranial ICA	9 (7.63%)	5 (5.32%)	4 (16.67%)	
Tandem lesion	14 (11.86%)	11 (11.70%)	3 (12.50%)	
M1	81 (68.64%)	65 (69.15%)	16 (66.67%)	
Proximal M2	14 (11.86%)	13 (13.83%)	1 (4.17%)	
*TOAST type*				0.05
LAA	51 (43.22%)	43 (45.74%)	8 (33.33%)	
Cardioembolism	49 (41.53%)	41 (43.62%)	8 (33.33%)	
other etiology	11 (9.32%)	6 (6.38%)	5 (20.83%)	
small-vessel	1 (0.85%)	1 (1.06%)	0 (0%)	
undetermined etiology	6 (5.08%)	3 (3.19%)	3 (12.50%)	
NIHSS score	12.00 (7.00, 17.00)	12.50 (7.75, 17.00)	11.00 (5.25, 16.00)	0.58
*Tan score*				0.86
0	4 (3.39%)	3 (3.19%)	1 (4.17%)	
1	35 (29.67%)	28 (29.79%)	7 (29.17%)	
2	50 (42.37%)	41 (43.62%)	9 (37.50%)	
3	29 (24.58%)	22 (23.40%)	7 (29.17%)	
*Menon score*				0.98
0	4 (3.39%)	3 (3.19%)	1 (4.17%)	
1	12 (10.17%)	9 (9.57%)	3 (12.50%)	
2	12 (10.17%)	10 (10.64%)	2 (8.33%)	
3	35 (29.66%)	28 (29.78%)	7 (29.17%)	
4	34 (28.81%)	28 (29.78%)	6 (25.00%)	
5	21 (17.80%)	16 (17.02%)	5 (20.83%)	
rLMC score	14.50 (10.00,17.00)	14.50 (10.00–17.00)	13.45 ± 5.01	0.99

Continuous variables were presented as mean ± standard deviation or median (interquartile range). Categorical variables were expressed as frequency and percentage. ICA, internal carotid artery; TOAST, trial of Org10172 in acute stroke treatment; LAA, large-artery atherosclerosis; NIHSS, National Institute of Health stroke scale; rLMC, regional leptomeningeal collateral.

### 3.2 The performance of our AI model compared with GT

**Figure 3:**
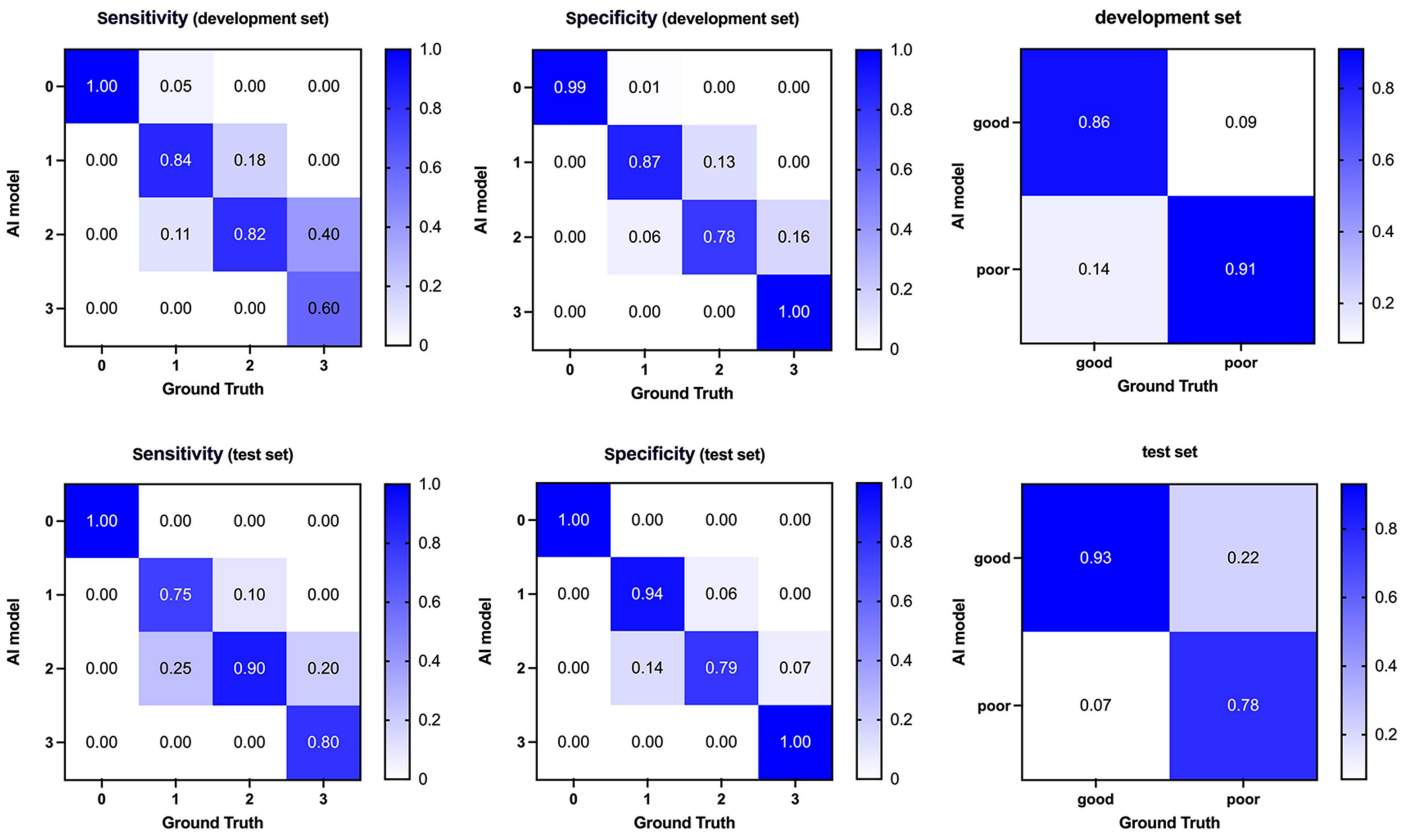
Confusion matrices and performance of our AI model compared with ground truth on the development (top) and test (bottom) dataset. AI, Artificial Intelligence.

**Figure 4:**
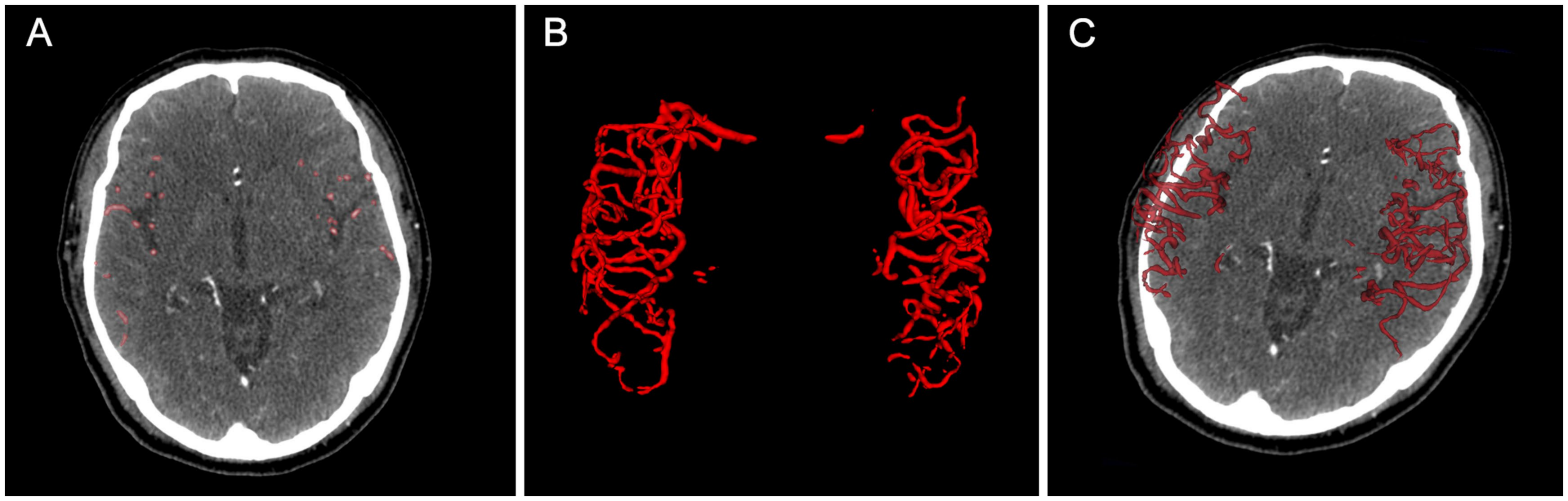
An example of collateral circulation quantification using our AI model. **(A)** shows AI-predicted vessel segmentations on axial CTA images, while **(B,C)** display 3D visualizations. The case achieved a DSC of 0.80 and a qCS of 88.49%, closely matching the GT (90.99%). AI, Artificial Intelligence; CTA, computed tomography angiography; DSC, Dice Similarity Coefficient; qCS, quantitative Collateral Score; ground truth, GT.

Table 2 and Figure 3 comprehensively present the performance metrics of our model, while Figure 4 provides an illustrative case to further demonstrate its capabilities. Our model exhibited robust image segmentation capabilities, achieving a mean DSC of 0.72 for the development set and 0.75 for the test set. Notably, the mean qCS derived from our AI model yielded values of 63.80% for both development and test cohorts, closely approximating but slightly lower (by approximately 5%) than the GT values, which were 69.01% for the development set and 69.11% for the test set.

**Table 2:** Classification performance of our AI model.

Characteristics	Development set (*n* = 94)	Test set (*n* = 24)
Compared to GT		
Four-class		
Accuracy	0.80	0.83
Two-class		
Accuracy	0.87	0.88
F1 score	0.91	0.90
MCC	0.70	0.73
AUC	0.89 (0.80–0.97) *p* < 0.001	0.86 (0.68–1.00) *p* = 0.004
Compared to Tan		
Four-class		
Accuracy	0.72	0.71
Two-class		
Accuracy	0.87	0.83
F1 score	0.91	0.88
MCC	0.71	0.63
AUC	0.84 (0.74–0.94) *p* < 0.001	0.81 (0.61–1.00) *p* = 0.014

AI, artificial intelligence; GT, ground truth; MCC, Matthews correlation coefficient; AUC, area under the receiver operating characteristic curve.

Regarding classification accuracy, our model achieved 0.80 and 0.83 for the four-class output in the development and test sets, respectively. Notably, when transitioning to binary-class outputs, these accuracies underwent a marked improvement, reaching 0.87 and 0.88, respectively. In the binary classification context, our model demonstrated a sensitivity of 0.86 and 0.93 for the development and test sets, respectively. Furthermore, the F1 score, which indicates a strong balance between precision and recall, ranged from 0.90 to 0.91 across the development and test sets. Lastly, the AUC values of 0.89 and 0.86 achieved in the development and test sets, respectively, further validate the model’s effectiveness in discriminating between classes.

### 3.3 The performance of our AI model compared with vCS

**Figure 5:**
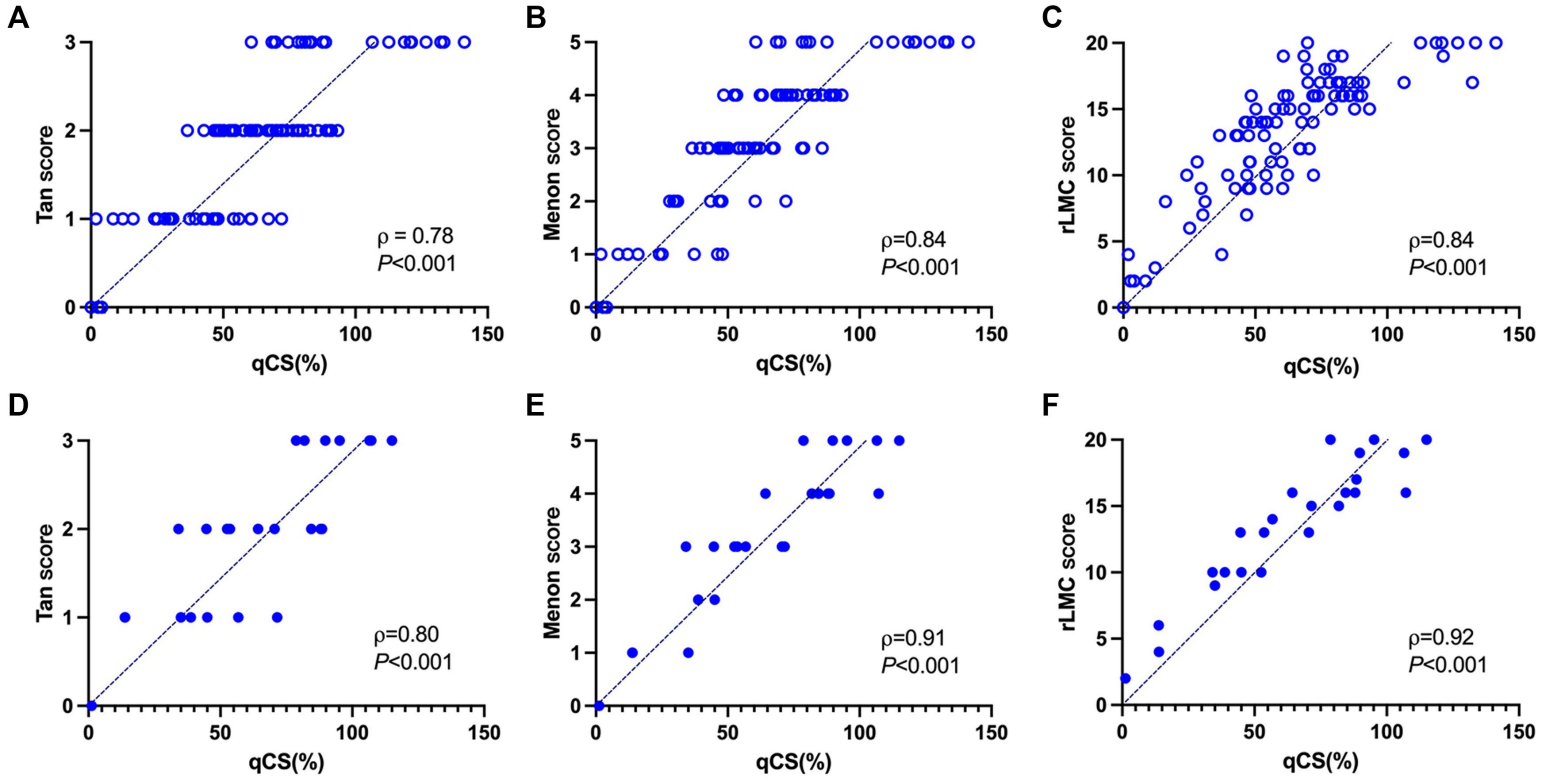
Correlation analysis between the qCS and three manual visual scoring methods in the development set **(A–C)** and test set **(D–F)**, respectively. qCS, quantitative collateral score; rLMC, regional leptomeningeal collateral.

The correlation analysis between the qCS and the manual visual assessments (Tan, Menon, and rLMC scores) is illustrated in Figure 5. The qCS demonstrated strong correlations with vCS, with *p* values ranging from 0.78 to 0.92 (all *p* < 0.001). It correlated even more strongly with the Menon score (development set: *ρ* = 0.84; test set: *ρ* = 0.91) and the rLMC score (development set: *ρ* = 0.84; test set: *ρ* = 0.92) than with the Tan score (*ρ* = 0.78 and 0.80, respectively for the development and test sets).

**Figure 6:**
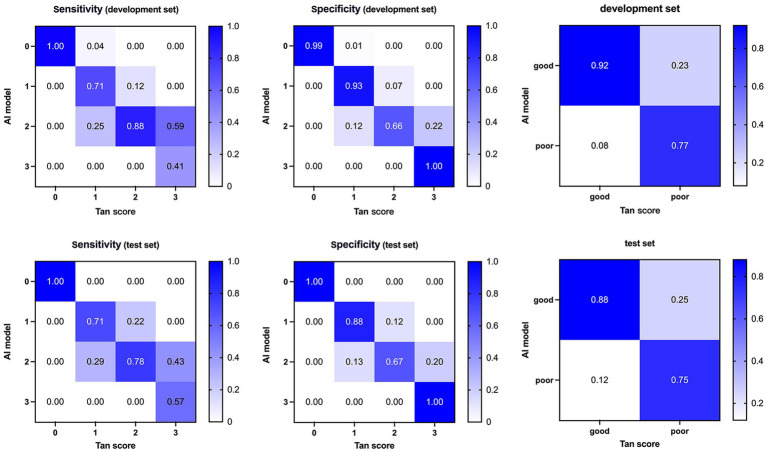
Confusion matrices and performance of our AI model compared with visual Tan score on the development (top) and test (bottom) dataset. AI, Artificial Intelligence.

**Figure 7:**
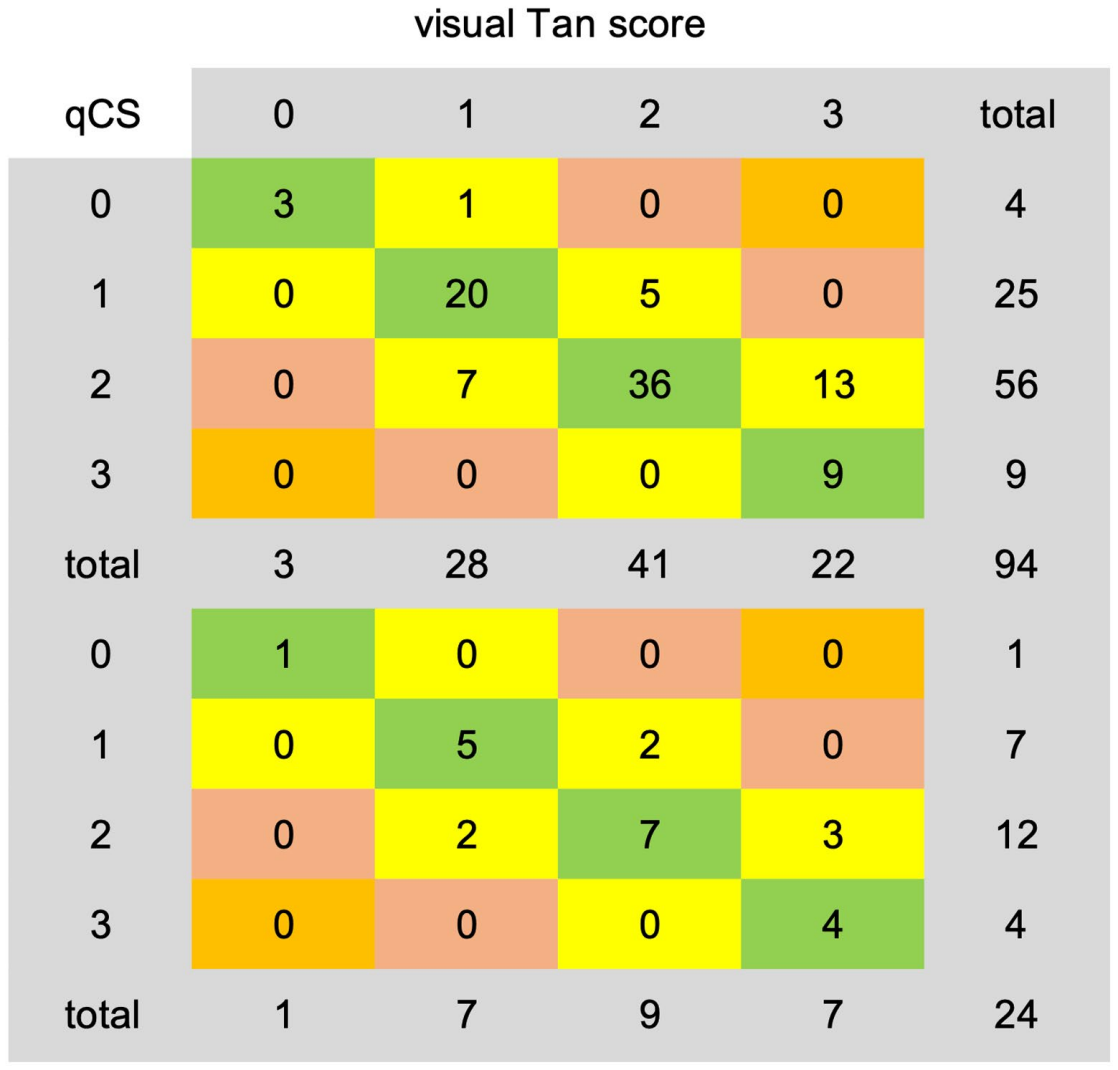
Agreement details between qCS-derived categorical results and visual Tan score on the development (top) and test (bottom) dataset. qCS, quantitative collateral score.

The categorical results derived from qCS demonstrated stronger agreement with the Menon score (development set: ICC = 0.83, *p* < 0.001; test set: ICC = 0.93, *p* < 0.001) compared to the Tan score (development set: ICC = 0.76, *p* < 0.001; test set: ICC = 0.79, *p* < 0.001). Table 2 and Figure 6 comprehensively encapsulate the model’s performance metrics, while Figure 7 elaborates on the class-by-class agreement specifics. When using the visual Tan score as the benchmark, qCS achieved an accuracy of 0.72 and 0.71 in the development and test cohorts, respectively, for a four-category classification, with a relatively low sensitivity in the grade of 3 (as depicted in Figure 6). Notably, in a binary classification framework, this accuracy increased to 0.87 (development set) and 0.83 (test set), respectively. Importantly, the discrepancies between the qCS-based scoring and the Tan score were minimal, confined within a 1-point margin, with no instances exceeding a 2-or 3-point difference (see Figure 7).

In the development set, the AI model exhibited underestimation in 19/94 (20.21%) cases and overestimation in 7/94 (7.45%) cases. Analogously, in the test set, underestimation was observed in 5/24 (20.83%) cases, while overestimation amounted to 2/24 (8.33%) cases. However, when adopting a Tan score threshold of ≥2 as indicative of favorable collateral circulation, the underestimation rate was remarkably decreased, misclassifying only 5/94 (5.32%) cases in the development set and 2/24 (8.33%) cases in the test set into the poor category.

## 4 Discussion

In the current study, we have successfully employed the deep learning framework of U^2^-Net to derive a quantitative collateral index, qCS, which exhibits strong correlation and excellent agreement with manual visual assessment, as evidenced by the high correlation coefficients ranging from 0.78 to 0.92 and ICCs spanning 0.76 to 0.93. This pivotal finding underscores the immense potential of AI-driven quantification in furnishing reliable and objective metrics for the assessment of cerebral collateral circulation.

Previous endeavors in quantifying collateral status have yielded promising results, albeit with varying degrees of accuracy. Boers et al.’s groundbreaking work, which correlated their qCS derived from Hessian-based enhancement filters with the visually determined Tan score, yielded a significant strong association (*ρ* = 0.75, *p* < 0.001) (16), mirroring our own findings (*ρ* = 0.78, *p* < 0.001). This convergence underscores the reliability of computational methods in mirroring expert visual assessments. Building upon this progress, Wolff et al. (17) innovated by upgrading and integrating this algorithm into a Web-based AI platform, StrokeViewer, intended for broader multi-center applications. However, when scaled up, their results revealed limitations in scalability and potential variability across institutions, as evidenced by a moderate inter-rater agreement (ICC = 0.60) and a modest four-class accuracy of 0.59. In contrast, our study surpasses these benchmarks, achieving a higher ICC of 0.79 and a more robust four-class accuracy of 0.71. This enhanced performance points to a reduced bias in our AI model, though it is pertinent to acknowledge that our single-center design may have contributed to these favorable outcomes.

Su et al. (18) employed a 3D U-Net to quantify collateral circulation. Although their model achieved a respectable binary accuracy of 0.90, the average DSC of 0.56 indicates room for improvement in segmentation precision. In response, our U^2^-Net model, with a DSC of 0.75, outperforms by incorporating pooling in residual U-blocks, enhancing network depth and feature extraction, leading to more accurate segmentation of collateral vessels. Turning to alternative input images, several studies used MIP images for collateral assessment to avoid manual annotation. For example, Fortunati et al. (19) used a Siamese model achieving a four-class classification accuracy of 0.64 and a two-class classification accuracy of 0.86. Kuang et al. (8) presented a hybrid CNN and Transformer model, MPViT, demonstrating a robust ICC of 0.77 for a three-point collateral score classification but a relatively low sensitivity of 0.61 in dichotomized analysis, Aktar et al. (20) found that EfficientNet B0 shines in 2-class classification but faces sample size constraints (n = 83). Although these models represent significant strides in collateral classification, they are unable to produce specific quantified results, which is a pivotal step towards a deeper comprehension of collateral status and its influence on patient outcomes.

In this study, qCS showed stronger correlation and higher concordance with more refined Menon score (test set: *ρ* = 0.91, ICC = 0.93) than with Tan score (test set: *ρ* = 0.80, ICC = 0.79), underscoring the precision of our assessment. This disparity further highlights the potential limitations of the Tan score’s four-category system, which employs relatively wide intervals (50% between classes), resulting in a coarser assessment. In contrast, the Menon and rLMC scores encompass a broader point range and finer grading, thereby offering a more precise predictor of imaging and clinical outcomes. Additionally, Yang et al. (21) also compared the qCS against both the conventional Tan’s score and a refined six-category Tan’s score (based on 25% intervals), finding a slightly higher correlation with the refined version (*ρ* = 0.78) than the conventional (*ρ* = 0.76), echoing our findings. Our results further emphasize that while the Tan score offers simplicity and convenience, its validity is compromised. Despite demonstrating stronger correlations with qCS, the intricate assessment procedures of the Menon and rLMC scores pose accessibility and feasibility challenges, making them most suitable for experienced neuroradiologists. Therefore, the development of our model aims to enhance the accuracy of collateral assessment among physicians, particularly those in early career stages.

Our AI model, when generating four-category results, exhibits a tendency to underestimate collateral circulation, frequently misclassifying scores of 3 as 2, resulting in a lower sensitivity for score 3 classifications. Nevertheless, this bias does not significantly alter clinical decision-making as a score of 2 still signifies robust collaterals, maintaining the viability of endovascular therapy. Conversely, in binary outcomes, the model tends towards overestimation, ensuring that more patients are considered for endovascular therapy eligibility by minimizing the risk of exclusion. Notably, Fortunati et al. (19) also observed their model’s greatest confusion resided between scores 2 and 3, with minimal ambiguity between 0 and 1. Mair’s evaluation of commercial software e-CTA reported similar underestimation (6%) and overestimation (7%) rates, with the underestimation closely aligning with our results (5–8%) (22). Su et al. (18), leveraging the visual Tan score as a benchmark, achieved an 80% accuracy in four-valued classification and a remarkable 90% in binary classification. The substantial boost in binary accuracy suggests a primary discrepancy arising from the Tan score range, particularly between scores 0–1 and 2–3. Similarly, in our study, we observed a remarkable surge in binary classification accuracy, a 12.50% increase, primarily due to underestimating Tan score 3 cases as 2. This phenomenon may stem from the inherent difference between the precision of qCS and the subjective nature of visual assessments, as the 95% threshold utilized by qCS may not adequately differentiate between visual scores 2 and 3.

Our study had some limitations. First, artificial bias may be introduced in the annotation of collateral vessels. To minimize this bias, repeated training and practice were conducted before annotation until the approval from the senior neuroradiologist. Second, its single-center design and lack of external validation may limit the generalizability and robustness of the findings. Third, the model demonstrates reduced sensitivity for 3-point collateral scores. This limitation could be addressed in future studies by exploring alternative network architectures and expanding the training dataset with additional 3-point cases, potentially through multicenter collaborations, to improve model generalizability. Finally, the relationship between qCS and clinical treatment decision as well as patient outcomes remains underexplored, necessitating further research to fully elucidate their significance.

## 5 Conclusion

In conclusion, our U^2^-Net model, designed to predict collateral scores from acute stroke CTA images, achieves exceptional segmentation performance with a DSC of 0.75. The qCS index shows strong agreement with visual assessment (accuracy = 0.83), establishing the technical feasibility of automated collateral quantification. While further validation is required to determine clinical utility, this framework provides a methodological foundation for subsequent research exploring quantitative collateral metrics in treatment decision-making and outcome prediction.

## Data Availability

The raw data supporting the conclusions of this article will be made available by the authors, without undue reservation.
