# Supplementary material for: Automatic collateral quantification in acute ischemic stroke using U2-net
Source: Front Neurol. 2025 May 12;16:1502382. doi: 10.3389/fneur.2025.1502382 (PMC12104720; doi:10.3389/fneur.2025.1502382)
Supplement: Supplementary file 1 [file Table_1.DOCX]

**Supplementary File 1**

**Table 1. Thresholds selection**

| qCS(%) | **Development cohorts** | | |  | **Test cohorts** | | |
| --- | --- | --- | --- | --- | --- | --- | --- |
|  | ACC | SEN | SPE |  | ACC | SEN | SPE |
| **0** | **0.9787** | **0.3333** | **1.000** |  | **0.9583** | **0.0** | **1.0** |
| 1 | 0.9787 | 0.3333 | 1.000 |  | 0.9583 | 0.0 | 1.0 |
| 2 | 0.9681 | 0.3333 | 0.989 |  | 1.000 | 1.0 | 1.0 |
| 3 | 0.9787 | 0.6667 | 0.989 |  | 1.000 | 1.0 | 1.0 |
| 4 | 0.9787 | 0.6667 | 0.989 |  | 1.000 | 1.0 | 1.0 |
| 5 | 0.9894 | 1.0000 | 0.989 |  | 1.000 | 1.0 | 1.0 |
| 6 | 0.9894 | 1.0000 | 0.989 |  | 1.000 | 1.0 | 1.0 |
| 7 | 0.9894 | 1.0000 | 0.989 |  | 1.000 | 1.0 | 1.0 |
| 8 | 0.9894 | 1.0000 | 0.989 |  | 1.000 | 1.0 | 1.0 |
| 9 | 0.9787 | 1.0000 | 0.978 |  | 1.000 | 1.0 | 1.0 |
| 10 | 0.9787 | 1.0000 | 0.978 |  | 1.000 | 1.0 | 1.0 |
| 45 | 0.8298 | 0.5484 | 0.9683 |  | 0.7917 | 0.625 | 0.8750 |
| 46 | 0.8298 | 0.5484 | 0.9683 |  | 0.8333 | 0.750 | 0.8750 |
| 47 | 0.8511 | 0.6452 | 0.9524 |  | 0.8333 | 0.750 | 0.8750 |
| 48 | 0.8723 | 0.7419 | 0.9365 |  | 0.8333 | 0.750 | 0.8750 |
| 49 | 0.8723 | 0.7742 | 0.9206 |  | 0.8333 | 0.750 | 0.8750 |
| **50** | **0.8617** | **0.7742** | **0.9048** |  | **0.8333** | **0.750** | **0.8750** |
| 51 | 0.8511 | 0.7742 | 0.8889 |  | 0.8333 | 0.750 | 0.8750 |
| 52 | 0.8511 | 0.7742 | 0.8889 |  | 0.8333 | 0.750 | 0.8750 |
| 53 | 0.8404 | 0.7742 | 0.8730 |  | 0.7917 | 0.750 | 0.8125 |
| 54 | 0.8404 | 0.8065 | 0.8571 |  | 0.7500 | 0.750 | 0.7500 |
| 55 | 0.8298 | 0.8387 | 0.8254 |  | 0.7500 | 0.750 | 0.7500 |
| 90 | 0.8298 | 0.4091 | 0.9583 |  | 0.8750 | 0.5714 | 1.0 |
| 91 | 0.8511 | 0.4091 | 0.9861 |  | 0.8750 | 0.5714 | 1.0 |
| 92 | 0.8511 | 0.4091 | 0.9861 |  | 0.8750 | 0.5714 | 1.0 |
| 93 | 0.8511 | 0.4091 | 0.9861 |  | 0.8750 | 0.5714 | 1.0 |
| 94 | 0.8617 | 0.4091 | 1.000 |  | 0.8750 | 0.5714 | 1.0 |
| 95 | 0.8617 | 0.4091 | 1.000 |  | 0.8750 | 0.5714 | 1.0 |
| 96 | 0.8617 | 0.4091 | 1.000 |  | 0.8333 | 0.4286 | 1.0 |
| 97 | 0.8617 | 0.4091 | 1.000 |  | 0.8333 | 0.4286 | 1.0 |
| 98 | 0.8617 | 0.4091 | 1.000 |  | 0.8333 | 0.4286 | 1.0 |
| 99 | 0.8617 | 0.4091 | 1.000 |  | 0.8333 | 0.4286 | 1.0 |
| **100** | **0.8617** | **0.4091** | **1.000** |  | **0.8333** | **0.4286** | **1.0** |

Notes: The threshold of Tan score (0,50,100) is represented in bold underlined form. Red denotes the best thresholds.
